# Transcranial Direct Current Stimulation in Neuropathic Pain

**DOI:** 10.4172/2167-0846.S3-001

**Published:** 2013-04-21

**Authors:** Niran Ngernyam, Mark P Jensen, Narong Auvichayapat, Wiyada Punjaruk, Paradee Auvichayapat

**Affiliations:** 1 Department of Physiology, Faculty of Medicine, Khon Kaen University, Khon Kaen, Thailand; 2 Department of Rehabilitation Medicine, University of Washington, Washington, USA; 3 Department of Pediatrics, Faculty of Medicine, Khon Kaen University, Khon Kaen, Thailand

**Keywords:** Transcranial direct current stimulation, Neuropathic pain, Noninvasive brain stimulation

## Abstract

Neuropathic pain (NP) is one of the most common problems contributing to suffering and disability worldwide. Unfortunately, NP is also largely refractory to treatments, with a large number of patients continuing to report significant pain even when they are receiving recommended medications and physical therapy. Thus, there remains an urgent need for additional effective treatments. In recent years, nonpharmacologic brain stimulation techniques have emerged as potential therapeutic options. Many of these techniques and procedures – such as transcranial magnetic stimulation, spinal cord stimulation, deep brain stimulation, and motor cortical stimulation – have very limited availability, particularly in developing countries. Transcranial direct current stimulation (tDCS) is a noninvasive brain stimulation procedure that has shown promise for effectively treating NP, and also has the potential to be widely available. This review describes tDCS and the tDCS procedures and principles that may be helpful for treating NP. The findings indicate that the analgesic benefits of tDCS can occur both during stimulation and beyond the time of stimulation. The mechanisms of cortical modulation by tDCS may involve various activities in neuronal networks such as increasing glutamine and glutamate under the stimulating electrode, effects on the μ-opioid receptor, and restoration of the defective intracortical inhibition. Additional research is needed to determine (1) the factors that may moderate the efficacy of tDCS, (2) the dose (e.g. number and frequency of treatment sessions) that results in the largest benefits and (3) the long-term effects of tDCS treatment.

## Introduction

Neuropathic pain (NP) is pain that caused by damage to the central or peripheral nervous system or both [1]. There are numerous etiologies of nervous system injury including exposure to toxins, infection, viruses, metabolic disease, nutritional deficiencies, ischemia, trauma (surgical and nonsurgical), and stroke. Many conditions such as alcoholic polyneuropathy, chemotherapy-induced neuropathy, complex regional pain syndrome, diabetic peripheral neuropathy, entrapment neuropathies, phantom limb pain, post-herpetic neuralgia, and radiculopathy can cause peripheral NP; while a compressive myelopathy from spinal stenosis, myelopathy, multiple sclerosis pain, Parkinson disease pain, post-stroke pain, spinal cord injury pain, trigeminal neuralgia, and syringomyelia can be the causes of central NP [2].

NP is common, with estimation in the general population ranging from 3% to 18%, depended on the methods used to classify individuals as having this symptom [3]. Moreover, a lot of evidences support the conclusion that NP has a significant negative impact on the quality of life [4]. The treatments would ideally be based on the underlying causes and mechanisms of pain. Currently there are several therapeutic options for treating NP [5]. In 60% NP is still refractory to medical treatment [6]. In addition, there are many unanswered questions regarding the pathophysiology. One model hypothesizes that some NP conditions are the results of thalamic dysregulation, which inhibit the natural pain modulatory system thalamus [7, 8].

There are some pathophysiological mechanisms supposed to play role in NP such as: 1) peripheral sensitization-cellular mediators act to sensitize nociceptors to further neural input. This produces changes in the number and location of ion channels especially sodium channels in the injured nociceptor nerve fibers and their dorsal root ganglia. As a result, the threshold for depolarization is decreased and spontaneous discharges can occur in abnormal locations. Consequently, the response of nociceptors to thermal and mechanical stimuli is increased [2]. 2) central sensitization- prolonged release and binding of substances to neural receptors activate the N-methyl-D-aspartate (NMDA) receptor, which causes an increase in intracellular calcium levels which is considered important to maintain central sensitization. These changes lead to a series of biochemical reactions in dorsal horn neurons. The threshold for activation is decreased, the response to stimuli is increased in both magnitude and duration, and the size of the receptive field is enlarged. These changes result in an increased excitability and sensitivity of spinal cord neurons. Another central mechanism supposed to contribute to the development and maintenance of NP is called central disinhibition, which occurs when control mechanisms along inhibitory or modulatory pathways are lost or suppressed. This, in turn, results in abnormal excitability of central neurons [2], and 3) deafferentation- the injured nervous system circuitry is thought to generate aberrant nocioceptive impulses that are interpreted by the brain as pain. Thalamic integrative circuits may also behave as generators and amplifiers of nocioceptive signals. Sensory deafferentation after injured nervous system induces profound and long-lasting reorganization of the cortical and subcortical sensory maps in the brain. Pathophysiological consequences of such cortical plasticity may underlie the development of NP. Strategies aimed to reverse or to modulate the somatosensory neural reorganization after injury may be valuable alternative therapeutic approaches to NP [9].

Unfortunately, however, NP is highly refractory to treatment. The standard treatments are primarily pharmacological, such as antidepressants, antiepileptics, topical anesthetics, and opioids [10, 11]. Nonpharmacological treatment options include psychological approaches, physical therapy, interventional therapy and surgical procedures [5]. However, only 40% of cases obtained a favorable outcome from medications [10]. Moreover, many medications have negative side effects, including drowsiness, constipation, and dry mouth, which might cause termination of these treatments, even though they have benefits for reducing pain severity. Thus, many patients continue significant NP [12]. It could be very valuable as an alternative not only given its efficacy but also it is cheap and widely available treatment options. Therefore, establishing nonmedical, neuromodulatory approaches are promising [13]. Transcranial direct current stimulation (tDCS), an application of electrical currents to modify brain function is a very old technique, mentioned more than 200 years ago and was re-introduced for about 25 years. tDCS is a safe noninvasive technique in which a low amplitude electrical current is conducted to the cortex via scalp electrodes. There are two essential components to a tDCS device; the first is power supply and the second are electrodes. The power supply is nine volts of direct current, which is delivered via a pair of surface conductive electrodes. To decrease impedance, the electrodes are covered with saline or gel soaked sponges. The sizes of the electrodes used, which are suited for a constant current density and focality, are 25–35 cm^2^. The proper current density delivered is between 0.029 and 0.08 mA/cm^2^. The anode electrode carries the positive charge, and the cathode carries a negative charge. The effects on the activity or excitability of the neurons that lie directly under the electrodes differ as a function of the charge. Systematic animal studies in anesthetized rats demonstrated that weak direct currents, delivered by intracerebral or epidural electrodes, induce cortical activity and excitability diminutions or enhancements, which can be stable long after the end of stimulation. Subsequent studies revealed that the long-lasting effects are protein synthesis dependent and accompanied by modifications of intracellular cAMP and calcium levels. Thus, these effects share some features with the well-characterized phenomena of long-term potentiation (LTP) and long-term depression (LTD) [14]. Various parameters of tDCS have been used: stimulation sites including the motor cortex, dorsolateral prefrontal cortex, visual cortex, and the somatosensory cortex; intensities ranging from 1 to 5 mA; frequencies from single to repeated sessions on consecutive days; and stimulation durations of 5 to 30 minutes [15].

**Figure 1:**
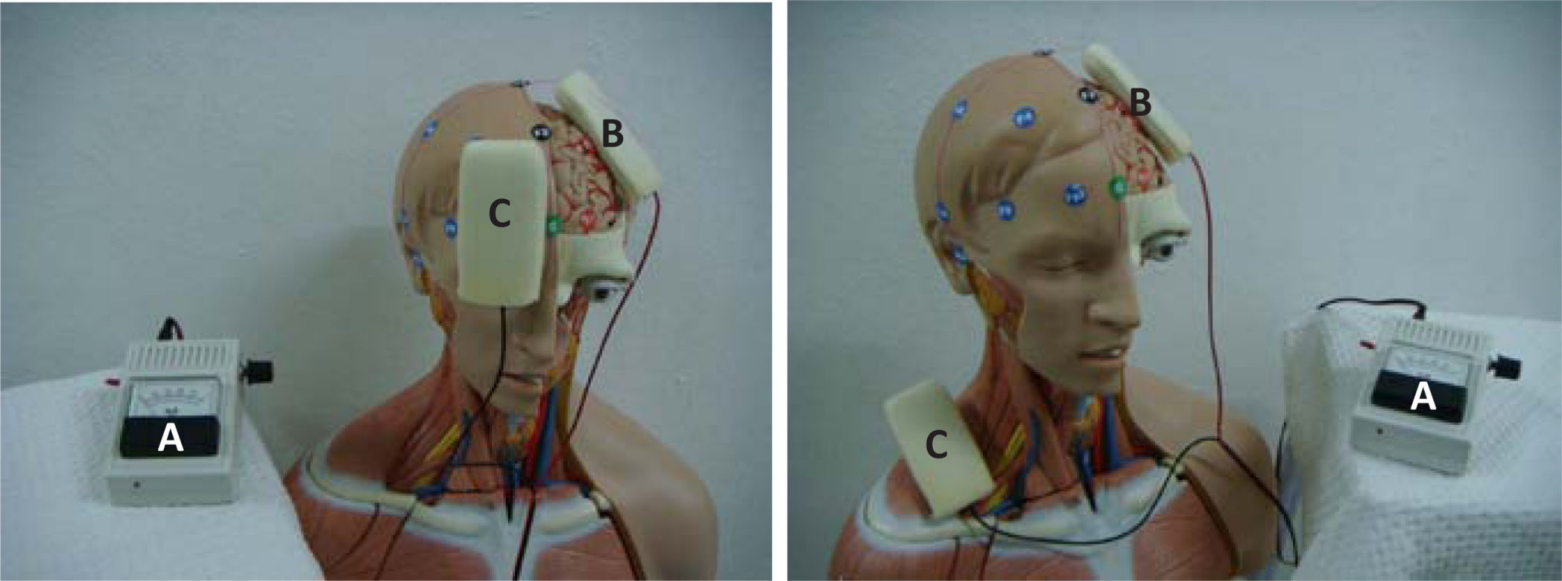
Transcranial direct current stimulation and 10–20 international electrode placements. A = Nine volts of direct current power supply, B = Stimulating electrode over the left primary motor area, and C = Reference electrode on the right supraorbital area or right shoulder area.

Some initial research in humans explored by Costain and coworkers of University College, London in 1964. They found that 2.5 mA anodal stimulation placed on the eyebrows was associated with decreasing in depressive symptoms [16], while cathodal stimulation in electrodes placed over the inner end of each eyebrow reduced manic symptoms [17]. Studies on the mechanism of action of tDCS showed that it causes polarity-dependent shifts of the resting membrane potential of the neurons that lie under the electrodes, consequently changing neuronal excitability at the site of stimulation and in connected areas [18]. In general, studies in humans have shown that the anode usually stimulates greater neuronal activity, that is why it is usually placed on the target area such as primary motor area (M1), central (C3 or C4), or frontal (F3 or F4) areas [12]. The reference electrode is usually placed on an extracephalic area such as contralateral supraorbital area or shoulder [19]. The locations of electrodes placing usually followed the international 10–20 electroencephalographic system as shown in the figure 1.

A number of previous studies have shown some promising beneficial effects of tDCS in the patients with NP [20–27]. Therefore the purpose of this review is to summarize what is currently known regarding the effects of tDCS on treatment of NP.

## Material and Method

A systematic review was conducted according to a predefined protocol and do not conduct a meta-analysis. Research studies examining the effects and mechanisms of tDCS on NP were identified via Medline database search using the key words “transcranial direct current stimulation and neuropathic pain” and “noninvasive brain stimulation and neuropathic pain” from January 1950 to January 2013. The trial intervention was defined as noninvasive electrical stimulation of the brain using direct currents. The inclusion criteria were: (a) the experimental studies; (b) case studies; (c) trial participants were adult patients with neuropathic or chronic pain (pain >3 mo). The exclusion criteria including: (a) healthy participants who were experimentally exposed to a pain paradigm; (b) studies on patients with primary symptoms other than pain, such as depression, stroke, or Parkinson disease; and (c) studies on surgically implanted brain stimulators, repetitive transcranial magnetic stimulation, and electrical stimulation with pulse currents. The outcome of interest was pain severity immediately after intervention as either the primary or secondary outcome parameter, the type of pain measurement tool was not predetermined.

## Results

A total of 20 studies identified by Medline searching, seven were excluded according to the exclusion criteria. We found seven studied on anti-neuropathic effect and six on possible mechanism of action.

### The clinical application of tDCS in anti-neuropathic effect

In 2006, Fregni and et al. [20, 21] published the first RCT to investigate the analgesic effect of 5 consecutive days of 2 mA anodal/sham tDCS (20 min per day) in 17 patients with refractory central pain following traumatic spinal cord injury (SCI) [20]. The anodal electrode was placed over the left or right M1 (contralateral to the pain area) and the cathode placed on the contralateral supraorbital area. Participants were randomized to receive active tDCS or sham tDCS. A 100 mm Visual Analog Scale (VAS) was used to evaluate pain intensity. They found that pain intensity was significantly (*p*=0.015) reduced in only treatment group at the third session, relative to baseline. Pain intensity continued to decrease until the fifth session (*p*=0.001). However, the pain reduction observed did not maintain at the 3-week follow-up. There was no change in cognitive function and no any serious side effects to the tDCS treatment. In control group, there was no statistically reduction in pain intensity every time points. In addition, the significant difference between active and control group was found in the second session (*p*=0.047) until fifth session (*p*=0.004) and the effect decreased in the last session as there was only a trend for difference between the two groups [20].

Fregni et al. then used the same protocol to examine the efficacy of tDCS in 32 patients with fibromyalgia patients [21]. Participants in this study were randomly assigned to one of three treatment conditions: (1) patients with anodal stimulation over primary motor cortex while the cathode was placed over the contralateral supraorbital area (M1 group); (2) patients underwent anodal stimulation over the left dorsolateral prefrontal cortex (DLPFC) while the cathode was placed on the contralateral supraorbital area (DLPFC group), and (3) patients in the sham group received sham stimulation of the primary motor cortex. The electrodes were placed over M1 for anodal stimulation, but the stimulator was turned off after 30 s of stimulation. They found that anodal tDCS in M1 group resulted in significantly greater pain reduction than either the DLPFC or sham stimulation (*p*<0.0001). The pain reductions observed peaked at the end of the week of stimulation, although it was still significantly less than baseline at the 3-week follow-up assessment (*p*=0.004). In addition, there was a small but statistically increase in quality of life measured by the Fibromyalgia Impact Questionnaire (FIQ) in M1 group than sham group (*p*=0.023) and the DLPFC group (*p*=0.018) [21].

Boggio et al. [22] completed a RCT using a crossover design to investigate the analgesic effect of a single session of active tDCS plus active TENS; active tDCS plus sham TENS; and sham tDCS plus sham TENS. The TENS electrodes were positioned 6 cm apart and centered over the site that elicited most pain on palpation during examination. The tDCS anodal electrode was placed over C3 or C4 and the cathode was placed on supraorbital region. The stimulated hemisphere was based on pain lateralization. With the tDCS treatment, a constant current of 2 mA was applied for 30 min. Assessments were performed immediately before and after each condition by a blinded rater. They revealed the statistically significant pain reduction in both (1) the active tDCS plus active TENS condition and (2) the active tDCS plus sham TENS condition (pain reduction 37%, *p*=0.004; 16%, *p*=0.014 respectively), but not the sham tDCS plus sham TENS condition (pain increase 2%, *p*=0.35) [22].

Mori et al. [24] tested the effectiveness of a 5 consecutive days of 2 mA anodal tDCS for 20 minutes over the M1 compared with sham tDCS [23]. They found a statistically significant pre-treatment to 4-week follow-up decreased in a VAS measure of pain intensity (37%) in 19 patients with chronic NP from multiple sclerosis (MS). Similarly, Antal et al. [23] found that the use of 4×4 cm^2^ electrodes in 12 patients with therapy-resistant chronic pain syndromes showed a great reduction of VAS after receiving tDCS than patients who received sham tDCS at day 28 after stimulation (*p*<0.05; VAS decreased in the active group = 27% and increased in the sham 6%, respectively). An examination of the course of pain reduction in the active group revealed that pain reduction began following the first stimulation (*p*=0.03). The maximal pain reduction occurred after the fifth stimulation (*p*=0.0006). At the follow up period, pain intensity increased slightly towards the pretreatment levels, although there was still a statistically significant reduction in pain intensity at the day 28 follow-up, relative to baseline (*p*=0.03). No patients reported severe adverse effects in this study, although seven patients reported suffered from light headache after anodal stimulation, and six reported a light headache after sham stimulation [24].

Soler et al. showed significant improvement in NP at 12 weeks after the combined anodal tDCS with walking visual illusion (VI) treatment, relative to participants in three control groups; anodal tDCS with control illusion, sham tDCS with VI, or sham tDCS with control illusion, in 39 patients with SCI and central NP [25]. In a subsequent study, Kumru et al. [26] examined 20 SCI patients without NP and 14 healthy subjects served as controls. Contact heat-evoked potentials (CHEPs) were recorded in response to stimuli applied at C4 level, and subjects rated their perception of evoked pain using a Numerical Rating Scale (NRS) (0 = no pain and 10 = unbearable pain) during CHEPs. Thirteen patients (65%) reported a mean decrease of 50% in the NRS for NP after tDCS plus VI. Moreover, evoked pain perception was significantly higher in the patients with NP than in the other two groups, but reduced significantly together with CHEPs amplitude after tDCS plus VI, relative to baseline. Pain perception thresholds were significantly lower before tDCS plus VI intervention than after treatment in the control group. The authors concluded that 2 weeks of tDCS plus VI induced significant changes in CHEPs, evoked pain and heat pain threshold in SCI patients with NP [26]. The application of tDCS researches in anti-neuropathic effect is summarized in table 1.

### Possible mechanisms of action

The mechanisms underlying the analgesic effects of tDCS are not fully understood. Researchers have suggested that tDCS could affect processing of activity in the motor [27], visual [28], somatosensory [29], prefrontal functions and systems [30], in addition to neuropathic pain [20–26].

One functional magnetic resonance imaging (fMRI) study revealed that 2.0 mA anodal tDCS applied over the left M1 activated not only the underlying cortex, but also the ipsilateral supplementary motor area and the contralateral posterior parietal cortex [31].

In addition, one MRS study applied 2.0 mA anodal tDCS for 30 min over P4, the right parietal cortex with the cathode placed on the contralateral arm. Significantly higher combined glutamate and glutamine levels were found beneath the stimulating electrode with non-significant increases in homologous regions of the opposite hemisphere [32]. Moreover, a significant interaction between hemispheres was found for tDCS effects on N-acetylaspartic acid, or N-acetylaspartate (NAA). These results suggest that changes in glutamatergic activity and tNAA may be related to the mechanisms by which tDCS influences learning and behavior [32]. These results provide support for the conclusion that tDCS results in effects that last beyond the treatment sessions, and may depend, at least in part, on synaptic plasticity changes related to transient activation of glutamatergic NMDA receptors [33, 34].

Recently, Portilla et al. showed the decreasing in intracortical facilitation, amplitude of motor evoked potentials, and an increase in intracortical inhibition in participants received a single session anodal tDCS over M1 [35]. They suggested that individuals with chronic NP may have defective intracortical inhibition.

Finally, DosSantos et al. identified μ-opioidergic effects of anodal tDCS by positron emission tomography (PET) [36]. They performed 2 mA anodal tDCS for 20 min in one trigeminal NP patient during PET scan using a μ-opioid receptor (μOR) selective radiotracer, [^11^C] carfentanil. The result showed the single active tDCS decreased μ-opioid receptor non-displaceable binding potential levels in (sub) cortical pain-matrix structures compared to sham tDCS, including nucleus accumbens, anterior cingulate cortex, insula, and especially in the posterior thalamus suggesting that the analgesic effect of M1-tDCS is possibly due to direct increase in endogenous opioid release. They also suggested that a single tDCS session may often have subclinical effects, but that repetitive sessions may be necessary to revert ingrained neuroplastic changes related to the chronic pain [36].

## Discussion

The evidence of anodal tDCS for NP reduction is based on 8 trials investigating clinical NP and 6 trials investigating the mechanism of NP relief. The clinical NP trials applied 1–2 mA anodal tDCS over the M1 or DLPFC for 20 minutes for 5 consecutive days. All authors reported significant duration of NP reduction between 2 and 4 weeks after treatment. No severe adverse effect was found in all of the studies.

The neurobiological effect of tDCS on NP suggested that individuals with chronic NP may have defective intracortical inhibition [35]. Because tDCS induces a weak, constant electric current, it has been proposed that anodal tDCS would cause antineuropathic effects by changing the membrane resting potential. On the other hand we can say that anodal tDCS would induce depolarization of the stimulated area [37]. In terms of the after-effects of stimulation, other mechanisms such as the synaptic transmission modulation via the NMDA receptors have been proposed and demonstrated experimentally [38]. Since tDCS seems to be able to change the state of local cortical excitability, this method might revert the dysfunctional brain activity changes associated with NP. As anodal stimulation increases cortical excitability, the improvement in pain after this treatment might have been related to an up-regulation of motor cortex activity leading to the modulate pain perception through indirect effects of neural networks on pain-modulating areas, such as thalamic nuclei. Past neuroimaging research has shown that stimulation of the motor cortex with epidural electrodes changes activity in thalamic and subthalamic nuclei [39]. A model has been proposed in which thalamic nuclei activation would lead to several events in other pain-related structures, such as the anterior cingulate, the periaqueductal gray, and the spinal cord, that could ultimately modulate the affective–emotional component of pain and also inhibit pain impulses from the spinal cord [40].

## Conclusion

This review supports the potential for tDCS to make significant reduction in NP, at least in the short term. The findings support the need for larger clinical trials that would help to determine (1) the ideal dose of tDCS (number and frequency of treatment sessions) for maximizing benefits, (2) how long the treatment benefits maintain and for how many patients, (3) whether or not “booster” sessions of tDCS might be needed to help maintenance of long-term benefits, and (4) how tDCS might best be combined with other treatments to maximize overall treatment efficacy for reducing pain and maximizing quality of life in individuals with NP.

## Figures and Tables

**Table 1:** Summary of tDCS in treatment of neuropathic pain [20–26].

Topic/ authors	Study design	Number of Subjects	Treatment	Stimulation electrode position	Reference electrode position	Stimulation Duration	Intensity (mA)	Results	Effective duration	Adverse events
Fregni et al. [20]	RCT in traumatic spinal cord injury	17	Anodal and Sham tDCS	Left or right M1	Contralateral supraorbital area	20 min (5 days)	2	Significant pain reduction after active tDCS(p<0.05), but not after sham stimulation	16 days	Mild headache and itching under the electrodes
Fregni et al. [21]	RCT in fibromyalgia	32	Anodal and Sham tDCS	Left or right M1, DLPFC	Contralateral supraorbital area	20 min (5 days)	2	Significant pain reduction in M1 than DLPFC and sham (p < 0.0001)	3 weeks	Sleepiness, itching, and headache (similar to sham group)
Boggio et al. [22]	RCT and cross over study in chronic pain	8	Active tDCS+active TENS, active tDCS+sham TENS, sham tDCS+shamTENS	tDCS over M1 TENS at the site of most pain	Contralateral supraorbital area	30 min (single stimulation)	2	Active tDCS+ active TENS induced greater pain reduction than others	N/A	N/A
Mori et al. [23]	RCT in chronic pain from multiple sclerosis	19	Anodal and sham tDCS,	M1	Contralateral supraorbital area	20 min (5 days)	2	Statistically decrease of pain scores at the end of stimulation	4 weeks	N/A
Antal et al. [24] (2010)	RCT in chronic pain	21	4×4 cm^2^ electrodes size of anodal and sham tDCS	M1	Contralateral supraorbital area	20 min (5 days)	1	-Pain reduction occurred after the first stimulation(p=0.03) -The maximal pain reduction occure at the 5^th^ stimulation	28 days	Light headache
Soler et al. [25]	RCT in central pain from spinal cord injury	39	Anodal tDCS+walking visual illusion, anodal tDCS+ control illusion, sham tDCS+visual illusion	M1	Contralateral supraorbital area	20 min (10 days)	2	Pain reduction was significant reduced in anodal tDCS+walking visual illusion group than others	12 weeks	N/A
Kumru et al. [26]	RCT in neuropathic pain from spinal cord injury	18	Anodal tDCS+visual illusion	M1	N/A	20 min	2	Evoked pain and heat pain threshold in spinal cord injury with neuropathic pain were significant changes after treated with anodal tDCS + visual illusion	2 weeks	N/A
